# Optimising the Management of Malignant Melanoma during COVID-19

**DOI:** 10.1016/j.jpra.2021.09.004

**Published:** 2021-11-08

**Authors:** C.M. Hurley, L. Wrafter, A. Dhannoon, H. Regan, P.J. Regan

**Affiliations:** 1 Department of Plastic & Reconstructive Surgery, University Hospital Galway, Newcastle Road, Galway, Ireland; 2 Royal College of Surgeons in Ireland, St Stephen's Green, Dublin, Ireland; 3 Medical Student

## Introduction

The coronavirus disease 2019 (COVID-19) continues to present challenges for healthcare systems. This has resulted in the pragmatic restructuring of plastic surgery units worldwide^1^. This includes changes to leadership, staffing capabilities, redeployment and upskilling, adjustments to elective activity, and transformed patient pathways. In March 2020, all non-urgent elective surgery was cancelled across the UK and Ireland indefinitely^2^. It is estimated that over 28 million elective surgical procedures have been cancelled worldwide during the peak of the pandemic in both public and private practice^3^^,^ ^4^. During this period, many units reported a significant fall in urgent melanoma referrals, which may lead to patients presenting with advanced disease, requiring more extensive surgery, and obtaining inferior long-term outcomes^5^^,^ ^6^. The authors of this paper sought to characterise their own experience of invasive and non-invasive melanoma during the COVID-19 pandemic, with insights into our units restructuring to manage the disease continually and effectively.

## Methods

A retrospective chart review was performed on all patients diagnosed with invasive and non-invasive cutaneous melanoma between March to December of 2019 (pre-COVID-19) compared to 2020 (COVID-19 pandemic) in a single plastic surgery unit in Ireland. These months were selected as the first regional lockdown commenced in March. There were no exclusion criteria. Patient demographics, referral sources, surgical procedures, tumour characteristics, radiological findings, oncological therapies, and follow-ups were recorded. All data were anonymised and stored in Microsoft Excel (Redmond, Washington, USA).

Statistical analysis was performed using R v 3.6.3 (R Foundation for Statistical Computing, Vienna, Austria). Counts and percentages were used to summarise the distribution of categorical variables. The mean ± standard deviation (SD) and the median/interquartile range (IQR) were used to summarise the distribution of continuous normal and non-normal variables, respectively. Chi-square test of independence was used to assess the association between categorical variables. Unpaired t-test and Mann–Whitney test were used to compare the distribution of normal and non-normal variables between groups, respectively. Hypothesis testing was performed at 5% level of significance.

## Results

A total of 589 patients were included in the study. Of these, 314 patients (53%) were diagnosed with invasive melanoma, compared to 275 patients (47%) diagnosed with non-invasive disease (Table 1). The mean age was statistically, significantly older in 2019 compared to 2020. The most common invasive subtype diagnosed was superficial spreading melanoma (*n*=165, 29%), followed by nodular (*n*=67, 12%), lentigo malignant melanoma (*n*=29, 5%), and acral melanoma (*n*=18, 3%). The majority of lesions were in the head and neck (*n*=248), the upper limb (*n*=126), and the lower limb (*n*=116). Interestingly, the majority of patients diagnosed in 2019 were referred by the general practitioner (*n*=231, 83%) compared to 61% (*n*=179) in 2020 during the COVID-19 pandemic. Overall, more patients were diagnosed with both invasive and non-invasive melanoma in 2020 than in 2019 (*p*<0.05). The time from referral to biopsy was significantly higher in 2020 (64 days) compared to 2019 (28 days) (*p*<0.05).

**Table 1:** Descriptive statistics of the study sample categorized by year of diagnosis

s	2019 (pre-COVID-19)	2020 (COVID-19)	*p*
	(*n*=277)	(*n*=312)	
**Age in years, m (range)**	68.5 (25 – 96)	63.1 (24 – 91)	<0.001
**Sex**			0.573
Male	137 (49.5%)	146 (46.8%)	
Female	140 (50.5%)	166 (53.2%)	
**Malignancy**			0.003
Non-invasive	148 (53.4%)	127 (40.7%)	
Invasive	129 (46.6%)	185 (59.3%)	
**Breslow thickness**	3.11 (3.65)	2.60 (3.16)	0.200
**SLNBx status**			<0.001
Negative	22 (44.0%)	68 (73.1%)	
Positive	28 (56.0%)	22 (23.7%)	
**Metastatic status**			0.188
No	261 (94.2%)	302 (96.8%)	
Yes	16 (5.78%)	10 (3.21%)	
**Deposit size in *mm***	3.68 (4.06)	6.09 (8.66)	0.304
**Extracapsular extension**			0.167
No	17 (81.0%)	10 (58.8%)	
Yes	4 (19.0%)	7 (41.2%)	
**Completion lymphadenectomy**			0.034
Not performed	137 (89.5%)	296 (94.9%)	
Performed	16 (10.5%)	14 (4.49%)	
Awaits surgery	0 (0.00%)	2 (0.64%)	
**Number of positive nodes**	4.19 (4.46)	2.38 (1.69)	0.166
**Largest node identified**	12.4 (9.03)	14.6 (16.8)	0.732
**BRAF**			0.097
Negative	45 (70.3%)	38 (69.1%)	
Positive	19 (29.7%)	13 (23.6%)	
**Perineural invasion**			0.695
Negative	107 (90.7%)	138 (88.5%)	
Positive	11 (9.32%)	18 (11.5%)	
**Lymphovascular invasion**			0.284
Negative	107 (89.9%)	134 (84.8%)	
Positive	12 (10.1%)	24 (15.2%)	
**Ulceration**			0.227
Negative	92 (77.3%)	110 (70.1%)	
Positive	27 (22.7%)	47 (29.9%)	
**Microsatellite**			1.000
Negative	79 (90.8%)	142 (90.4%)	
Positive	8 (9.20%)	15 (9.55%)	
**Mitosis**	1.60 (3.95)	2.29 (4.98)	0.062

In terms of tumour characteristics, including the tumour Breslow thickness (BT), ulceration, perineural, and lymphovascular invasion, microsatellites and mitotic rate were not statistically significant between the two groups. Of the sentinel lymph node biopsies performed, the rate of positivity was higher in 2019 at 56% (*n*=28) compared to 24% (*n*=22) in 2020. The deposit size and presence of extracapsular extension were not significant between the two groups. Finally, the number of patients diagnosed with metastatic disease was not statistically significant between the two groups (*p*=0.188).

## Discussion

The diminution of elective plastic surgical activity during the COVID-19 pandemic will continue to have a profound impact on the delivery of healthcare services worldwide. As the incidence of malignant melanoma continues to increase in the UK and Ireland, it has the highest increment compared to other malignancies in the past two decades^7^. Promt diagnosis and treatment are the core principle in the prevention of morbidity and mortality of the disease. Early observers during the pandemic noted significantly more aggressive disease following regional lockdowns with respect to previously controlled time periods^6^^,^ ^8^. This decrease is contrary to our units’ experience of both invasive and non-invasive melanoma during the COVID-19 pandemic thus far. We found that more patients were diagnosed with both non-invasive and invasive melanoma during the pandemic than previously.

The referral source pattern demonstrated in our results is a record of the regional lockdown effect; many people with signs and symptoms of skin cancer did not report to their general practitioner due to uncertainties surrounding COVID-19 transmission^5^. Urgent referral care pathways between primary and tertiary care centres were also disrupted, protecting the burden on hospital resources^5^. This change is reflected in the increase in time from referral to formal diagnosis in our cohort. In response to the international experience of COVID-19 and in anticipation of a significant burden on our healthcare system, the Irish government reached an early agreement with the private sector to continue elective surgery on an urgent basis in private hospitals^9^. In particular, access to local anaesthetic procedures was reliable, and elective local skin cancer cases were triaged to non-COVID-19 provider centres.

Our study demonstrated similar tumour characteristics, including BT, ulceration, and levels of perineural and lymphovascular invasion between the two cohorts. The sentinel node positivity rate was similar across both groups, and the rate of metastatic disease observed was homogenous. Westen *et al.* summarise an American experience, with significant increases in tumour thickness and ulceration and advanced tumour stages. Similar findings were exhibited in Spanish and Italian populations^8^^,^ ^10^. As reflected in UK plastic surgery departments, our unit adopted a prompt telemedicine service for patients^1^^,^ ^4^^,^ ^5^^,^ ^11^^,^ ^12^. This strategy allowed for minimal patient contact and fast, effective, and appropriate triage of skin cancers. This structure remains in place and represents a potentially permanent shift in practice. During the pandemic, a skin cancer nurse specialist maintained patient follow-ups and triaged a dedicated email service. Finally, our plastic surgery service continued to maintain a virtual complex skin cancer multidisciplinary team meeting during the pandemic, ensuring local clinical governance was adhered to in each clinical case.

Despite early international reports of the impact of the COVID-19 pandemic on the detection, management, and outcomes of invasive malignant melanomas, our study highlights that with a prompt restructuring of services, our successful management of skin cancer can persevere. While our study is limited in its capture of a short period of the COVID-19 pandemic, it records the most devastating stage and the months following. Although plastic surgery services must continue to anticipate greater caseloads due to the unpredictable nature of the virus, it is important that we reflect on our successes during the pandemic as well as forecasting a potentially difficult future with the ongoing COVID-19 pandemic.

## Disclosures

**Funding**: No funding was sought for the preparation of this manuscript.

**Conflict of Interest**: None

**Ethical Approval**: N/A
